# Premature Beats Rejection Strategy on Paroxysmal Atrial Fibrillation Detection

**DOI:** 10.3389/fphys.2022.890139

**Published:** 2022-04-01

**Authors:** Xiangyu Zhang, Jianqing Li, Zhipeng Cai, Lina Zhao, Chengyu Liu

**Affiliations:** State Key Laboratory of Bioelectronics, School of Instrument Science and Engineering, Southeast University, Nanjing, China

**Keywords:** paroxysmal atrial fibrillation, paroxysmal atrial fibrillation detection, premature beats, ECG, low complexity

## Abstract

Paroxysmal atrial fibrillation (PAF) may related to the risk of thromboembolism and is the most common cardiac risk factor of cryptogenic stroke (CS). Due to its paroxysmal characteristics, it is usually diagnosed by continuous long-term ECG. Patients with paroxysmal atrial fibrillation usually have premature beats at the same time which is easy to be confused with the rhythm of atrial fibrillation. Therefore, in this article, we designed a screening algorithm for single premature beat, multi premature beats, bigeminy and trigeminy premature beats, according to their rhythm characteristics to reduce false detection caused by premature beats during the PAF detection process. The proposed elimination method was verified on ECG segments with different types of premature beats, and tested on long-term ECG data of PAF patients. ECG segments of different kinds of premature beats were selected from MIT Atrial Fibrillation database (MIT-AFDB), MIT-BIH Arrhythmia database (MIT-AR) and wearable ECG data from the China Physiological Signal Challenge 2021 (CPSC 2021). The proposed method can effectively eliminate single premature beat segments with 99.5% accuracy, and it also can eliminate more than 95% of ECG segments with other types of premature beats. We designed PAF-score as a new index to evaluate the accuracy of detection, and we also calculate the misjudged and missed segments to comprehensively evaluate the PAF detection algorithm. The proposed method get a PAF-score of 0.912 on MIT-AFDB. The proposed method also has the potential to implant low computing power wearable devices for real-time analysis.

## 1 Introduction

Atrial fibrillation (AF) is the most common cardiac arrhythmia in clinical practice and is associated with increased morbidity and mortality that primarily occur as a result of complications (1). AF may lead to stroke and congestive heart failure (CHF) and increase the death rate for AF patients (Gillis et al., 2013; Odutayo et al., 2016). For instance, up to a third of strokes have no known cause—so-called embolic stroke of undetermined source (ESUS) (Attia et al., 2019). Many of these strokes are related to atrial fibrillation, which can be under detected due to its paroxysmal and often asymptomatic nature. Paroxysmal atrial fibrillation (PAF) may be associated with risks of stroke and thromboembolism similar to those for sustained AF, and many patients suffer significant morbidity (Attia et al., 2019). The hazards of Paroxysmal atrial fibrillation are large, and because their own characteristics need to perform multiple long-term electrocardiography (ECG), qualitative parity atrial fibrillation for patients. The occurrence of PAF often cannot be detected within the first 48 h of ambulatory ECG monitoring (Solomon et al., 2016). Therefore, it is necessary to design an accurate paroxysmal atrial fibrillation detection algorithm and eliminate the false alarms caused by other arrhythmia to reduce the workload of doctors.

The ECG in AF duration has two main characteristics: 1) the absence of *p* waves and presence of undulating atrial activity, also known as fibrillatory waves or f waves. 2) highly irregular variation of RR intervals (Clifford et al., 2017; Platonov and Corino, 2018; Hayano et al., 2019). Most AF detection methods in previous literature was designed based on these two aspects. RR-intervals based classification method usually extracted features from RR intervals and use machine learning methods as classifiers, or use deep-learning based classification model and use RR interval sequences as input data directly (Lee et al., 2012; Zhou et al., 2014; Xiong et al., 2017; Dharmaprani et al., 2018; Kumar et al., 2018; Liu et al., 2018). Lake (Lake and Randall Moorman, 2011) verified that the coefficient of sample entropy (COSEn) of 12 RR intevals can accurately distinguish atrial fibrillation from normal ECG. Dash (Dash et al., 2009) calculate the randomness, variability and complexity of the RR intervals and use turning points ratio combination with the root mean square of successive RR differences and Shannon entropy to characterize AF. Faust used LSTM based deep learning model and used RR interval as input data to detect AF (Faust et al., 2018). Some deep-learning based methods also convert the ECG signal to a 2D representation. Xia et al. applied short-term Fourier transform (STFT) and stationary wavelet transform (SWT) to obtain the 2D matrix input suitable for deep 2D CNN models (Xia et al., 2018). Qayyum et al. converted ECG signals into 2D images by STFT, and used pre-trained CNN models for transfer learning (Qayyum et al., 2018). Lorenz plot imaging of ECG RR intervals was also used as input images to training a 2D CNN based model for AF classification (Hayano et al., 2019).

However, these method in previous literature usually divides the ECG signal into segments according to a certain length of time or certain length of RR intervlas (Kiranyaz et al., 2016; Chang et al., 2018; Tan et al., 2018; Kim and Pan, 2019; Yildirim et al., 2019). And then these segments are detected and classified as atrial fibrillation and non-atrial fibrillation. Most AF detection based on deep learning must require a fixed length of input data (Qayyum et al., 2018; Xia et al., 2018). Some AF detection devices are also designed to collect ECG signals for a specific length of time. Haberman (Haberman et al., 2015) detects atrial fibrillation by collecting a patient’s 30-s lead I ECG waveform using an iPhone case or iPad. Brasier acquire 1 min or 5 min ECG recordings for AF detection by smart-phones (Brasier et al., 2019). All of these methods are effective in detecting patients with permanent atrial fibrillation. However, there were usually premature beats in the ECG segments of PAF patients, which may result in some non-AF segments containing premature beats being misidentified as premature beats. These methods need to be further test of their ability to accurate classify the ECG segments containing premature rhythms.

In this paper, we designed a screening algorithm for single premature beat, frequent premature beats, bigeminy and trigeminy premature beats, according to their rhythm characteristics to reduce false alarms caused by premature beats during the PAF detection process. And we also selected ECG segments with these different types of premature beats from MIT-BIH Arrhythmia database (Moody and Mark, 2001), to verify the accuracy of the designed premature beat elimination algorithm. We designed PAF-score as a new index to evaluate the accuracy of detection and test the proposed PAF screening algorithm on MIT-BIH atrial fibrillation database (Moody and Mark, 1983).

## 2 Data

### 2.1 Definition of Different Premature Beats Types

**FIGURE 1:**
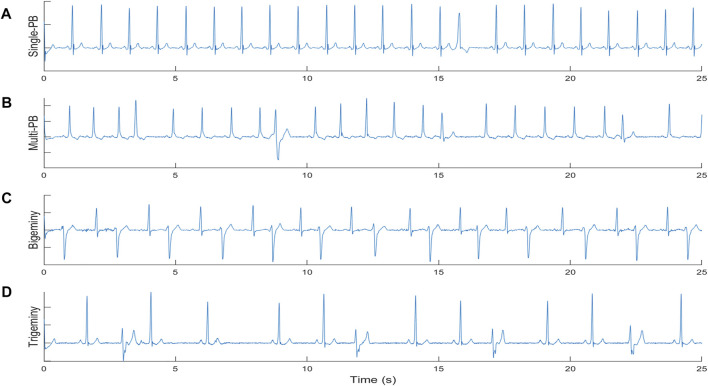
The definition of different premature beats types. **(A)** single premature beat; **(B)** multi premature beats; **(C)** Bigeminy; **(D)** Trigeminy.

In this paper, the proposed screening algorithm was designed for the rhythm characteristics of single premature beat, frequent premature beat, double premature beat, and triple premature beat. The definition of the four different premature beats types is as follow:

1) Single-PB: as shown in the sub-figure 1A of Figure 1, there was Only one premature beat in the ECG segment;
2) multi-PB: as shown in the sub-figure 1B of Figure 1, there were more than one premature beats in the ECG segment and the distribution of different premature beats is irregular;
3) Bigeminy: as shown in the sub-figure 1C of Figure 1, there were normal beats and premature beats appear alternately with more than six consecutive beats;
4) Trigeminy: as shown in the sub-figure 1D of Figure 1, there were two normal beats and premature beats appear alternately with more than six consecutive beats.

### 2.2 Database

#### 2.2.1 MIT-BIH Arrhythmia Database

The MIT-BIH Arrhythmia database (Moody and Mark, 2001) contains 48 half-hour excerpts of two-channel ambulatory ECG recordings, obtained from 47 subjects. The recordings were digitized at 360 samples per second per channel with 11-bit resolution over a 10 mV range. Two or more cardiologists independently annotated each record; disagreements were resolved to obtain the computer-readable reference annotations for each beat (approximately 110,000 annotations in all) included with the database. In this work, the ECG segments with heart beats marked as premature beats in the database was selected as test data to verify the accuracy of the designed premature beat elimination algorithm((Asgari et al., 2015; Ladavich and Ghoraani, 2015; García et al., 2016; Andersen et al., 2019))

The heart beats which was marked as premature beat (PB) in the annotation of the database and its surrounding heart beats were extracted as ECG segments with 31 beats (30 RR). Then the extracted segments were divided into four categories: Single premature beat (single-PB), multi premature beats (multi-PB), Bigeminy and Trigeminy.

1) Single-PB: Only one beat in the ECG segment is marked as PB;

2) multi-PB: The number of heart beats marked as PB in the ECG segment is more than 2;

3) Bigeminy: The ECG fragment contains the sequence “*N*N*N″ or “N*N*N*”;

4) Trigeminy: The ECG fragment contains the sequence “*NN*NN”, “N*NN*N″ or “NN*NN*“, where “*” indicates that the heartbeat is marked as PB, and the “N” mark means that the heartbeat is a normal heartbeat or other rhythms except PB and AF.

#### 2.2.2 MIT-BIH Atrial Fibrillation Database

In this study, we selected the MIT-AFDB as database which consists of 25 long term ECG recordings of human subjects with AF (mostly paroxysmal) (Moody and Mark, 1983). Each recording is 10-h duration, and contains two leads of ECG signals sampled at 250 Hz. The rhythm annotation files were prepared manually; they contain rhythm annotations of the following types, i.e., “AFIB” (atrial fibrillation), “AFL” (atrial flutter), “J” (AV junctional rhythm), and “N” (all other rhythms). In order to detect the start and end points of atrial fibrillation segments, the signals labeled as “AFIB” were used as the AF ECG samples and ECG signals labeled as other rhythm were referring to the non-AF ECG data. After this, these ECG recordings can be regarded as long-term ECG recordings composed of non-atrial fibrillation segments and atrial fibrillation segments connected to each other. The QRS detection method was performed on all recordings, and the detected beats were labeled to AF/non-AF according to the rhythm annotation. Thus, each segment of AF or non-AF can be composed of consecutive QRS waves of the same type, and the start and end points of each rhythm segment can be located on a certain QRS wave.

#### 2.2.3 China Physiological Signal Challenge 2021 (CPSC 2021)

The ECG data of CPSC 2021 are recorded from 12-lead Holter or 3-lead wearable ECG monitoring devices. The challenge ECG data provides variable-length ECG records fragments extracted from lead I and lead II of the long-term dynamic ECGs, each sampled at 200 Hz.

## 3 Methods

**FIGURE 2:**
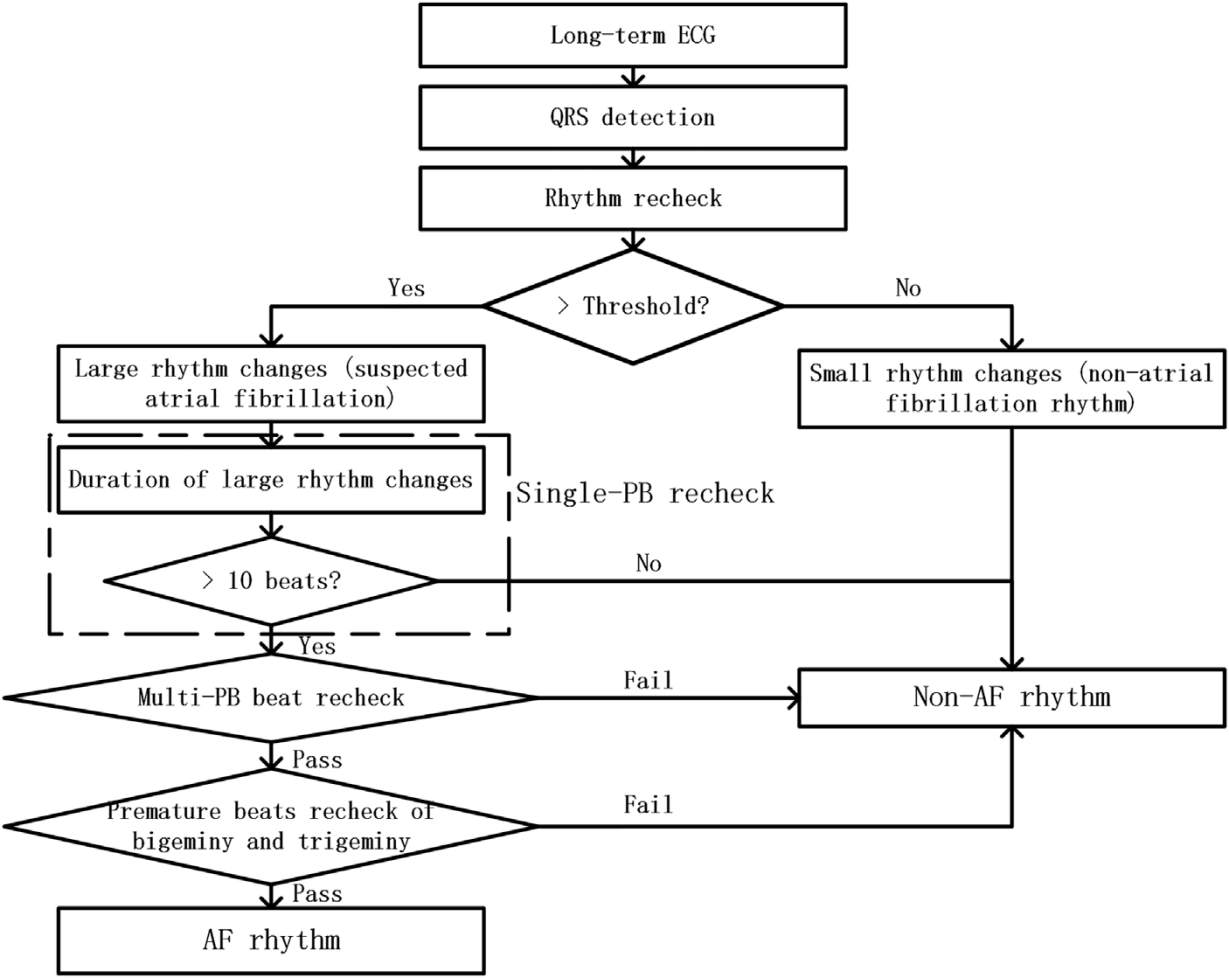
The flow chart of RR-interval based premature rhythms elimination method from PAF detection

As shown in Figure 2, the proposed PAF screening method is composed of three parts: pre-processing, suspicious AF segment screening, and premature beat screening method.

### 3.1 Pre-processing Method

In this study, ECG recordings were firstly remove their baseline drift through a sliding median filter. And QRS detection method was performed on the filtered ECG data. Then signal quality assessment method was utilized to remove the ECG segments with poor signal quality. The ECG segments with poor signal quality means that these ECG segments only contained noise without any ECG information. And the detected QRS locations in these bad quality were removed from the QRS sequences of the ECG recordings. Then, we fine-tune the detected QRS wave to ensure that the QRS wave is at the position of the maximum absolute value of the waveform in the neighboring area.

### 3.2 Suspicious AF Segment Screening Method

Threshold-based suspicious segment screening method was used to define the rhythm changes of the ECG segment. In order to assess the rhythm changes in a short period of time, we used the ratio of short-term RR interval’s standard deviation to its average value. For each QRS wave, we use six adjacent RR intervals to evaluate it is rhythm change. And the calculation method is shown in expression (Ogawa et al., 2018).

$$R_{c}=\frac{std\left(\left[RR1,RR2,\ldots ,RR6\right]\right)}{mean\left(\left[RR1,RR2,\ldots ,RR6\right]\right)}$$ (1)

where *R*_*c*_ refers to the rhythm change feature of the QRS. [*RR*1, *RR*2, … , *RR*6] refers to the array of 6 adjacent RR intervals after each QRS.

When the value of *R*_*c*_ exceeds the threshold, representing the difference between these RR intervals was large. Therefore, it is considered to have large rhythm changes. QRS segments which contained few rhythm changes in a short time were regard as non-AF segments and the QRS segments which contained large rhythm changes were regard as suspected AF segments. In this step, we remove the low rhythm change parts in the detected QRS sequence and the remaining QRS fragments will be further screened.

### 3.3 Premature Beats Reject Method

In this step, we mainly screen for ECG segments with premature beats that were easily confused with the atrial fibrillation rhythm. The rhythm recheck contains three screening aspects: single-PB recheck, multi-PB recheck, and premature beats recheck of bigeminy or trigeminy.

#### 3.3.1 Single Premature Beat Recheck

The effect of single-PB on the RR interval sequence is as follows: one smaller RR interval appears in the normal RR interval sequence, followed by one larger RR interval. Therefore, its impact on rhythm changes was relatively limited. From the first appearance of the small RR interval to the last appearance of the larger RR interval, the screening window with a length of 6 RRs slides Seven times. So theoretically, a single-PB usually only affects Rhythm assessment result for 7 *R*_*c*_ values of the consecutive RR intervals. Therefore, it is easy to filter out all single-PB by verifying whether the duration of continuous rhythm changes exceeds 10 beats.

For ECG segments passed single-PB recheck, their RR interval sequences were clustered into three categories by K-Medoids clustering algorithm. Each RR and the ratio of its first-order difference value to the RR were used as clustering features. And fine-tune the clustering results to reduce the standard deviation of the RR intervals within each group.

#### 3.3.2 Multi Premature Beats Recheck

Compared with single-PB, multi-PB have a higher probability of occurring in a short time, so the rhythm screening results will show continuous long-term large rhythm changes. However, when premature beats occur frequently, the proportion of normal heart beats is still the largest. Therefore, in order to reduce the influence of the abnormal RRs on the rhythm screening result, we selected the RR interval group with the closest mean RR interval to the median of the entire segment signal among the three categories, and then performed rhythm screening again. If the rechecked rhythm change screening result drops below the threshold, it means that the ECG segment being detected was with frequent premature beats.

#### 3.3.3 Premature Beat Recheck of Bigeminy and Trigeminy

Bigeminy and trigeminy are two special premature beats rhythm. Among them, the RR interval of bigeminy usually with one alternate change of long and short RR intervals, while trigeminy usually with one alternate change of three length RR intervals: short, long and normal. Therefore, when the number of larger and smaller RR intervals is consistent and both occurs more than two times in any 6 consecutive RRs, we believe that the ECG segments was with bigeminy or trigeminy rhythm. It is worth noting that, there was only little difference between the larger RR intervals in the ECG segments of bigeminy and trigeminy, so as the smaller RR intervals. Thus, we selected the larger RR intervals group of the clustering results, and then performed rhythm screening on the selected RR intervals. Then, the bigeminy and trigeminy premature beats can be removed from the suspected AF segments.

## 4 Result

### 4.1 Classification Result of ECG Segments With Premature Beats

The four different premature beat rhythm ECG segments were classified by the proposed elimination method, and the classify accuracy (*A*_*cc*_), error rate (*E*_*r*_) of the proposed method was showed in Table 1. The proposed method can eliminate 96.83% of the ECG segments with premature beat. Although the rigorous screening method resulted in 2.83% of the 3,000 test af ECG segments being erroneously eliminated, the overall accuracy of the proposed method in the 6,000 fragments also reached 97.00%. Moreover, the proposed method can eliminate ECG segments with single-PB with 99.5% accuracy.

**TABLE 1:** Result of ECG segments with four different kinds of premature beats.

Type	Total number	Classified	*A*_*cc*_(%)	*E*_*r*_ (%)
Single-PB	1,000	995	99.50	0.50
Multi-PB	1,000	957	95.70	4.30
Trigeminy	500	476	95.20	4.80
Bigeminy	500	477	95.40	4.60
AF segments	3,000	2,915	97.17	2.83
total	6,000	5,820	97.00	3.00

### 4.2 Result of PAF Detection

**FIGURE 3:**
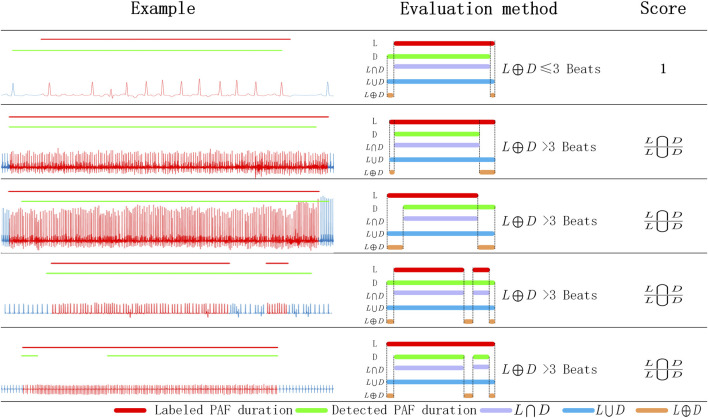
The evaluation scheme of PAF detection result. L: labeled PAF duration; D: detected PAF duration; *L*⋂*D*: the intersection of L and D; *L*⋃*D*: the union of L and D; *L⊕D*: the exclusive-OR of L and D.

To evaluate the PAF detection capability of the proposed method, we designed an PAF evaluation score (PAF-score) based on the annotated PAF time and the detected PAF time. We evaluate each PAF segment in the ECG records and give evalution score between 0 and 1. For each recording, its PAF-score was calculated as the average score of the annotated paf segments. As shown in Figure 3, only the difference between the labeled PAF time and the detected PAF time less than three heart-beats, it was considered that the detection result is consistent with the annotation and get the maximum score 1. Otherwise, it is considered that there is a non-negligible difference between the detection result and the annotated PAF time. And the score of these segments was calculated by the intersection and the union of the detected PAF time and the annotated PAF time. As shown in Table 2, the proposed method get a average PAF-score 0.912 on MIT-AFDB.

**TABLE 2:** Result of the proposed method test on MIT-AFDB.

Patients	Labeled PAF Segments	DetectedSegments	Error Segments	Missed Segments	Min Score	Max Score	Average Score
04,043	82	90	6	4	0.352	0.999	0.856
08,219	39	34	2	3	0.531	0.996	0.863
04,936	36	82	5	1	0.267	0.998	0.832
06,426	26	25	0	2	0.673	1	0.904
Total	278	356	33	21	-	-	0.912

This PAF-score is intended to reflect the accuracy of the algorithm for PAF segments detection. In order to comprehensively evaluate the performance of the algorithm, we have also counted the misjudgment segments and missing segments of the detection algorithm. The error segments in Table 2 means that the detected segments whithout QRS which were annotated as PAF rhythm. The missed segments refer to the labeled PAF segments which was completely detected as non-af rhythm. The proposed PAF detection method get an accuracy of 96.87% on the 23 recordings of MIT-AFDB. And the sensitivity and specificity of the proposed method were 96.43 and 97.24%, respectively.

## 5 Discussion

### 5.1 Suspicious AF Segment Screening Method

**FIGURE 4:**
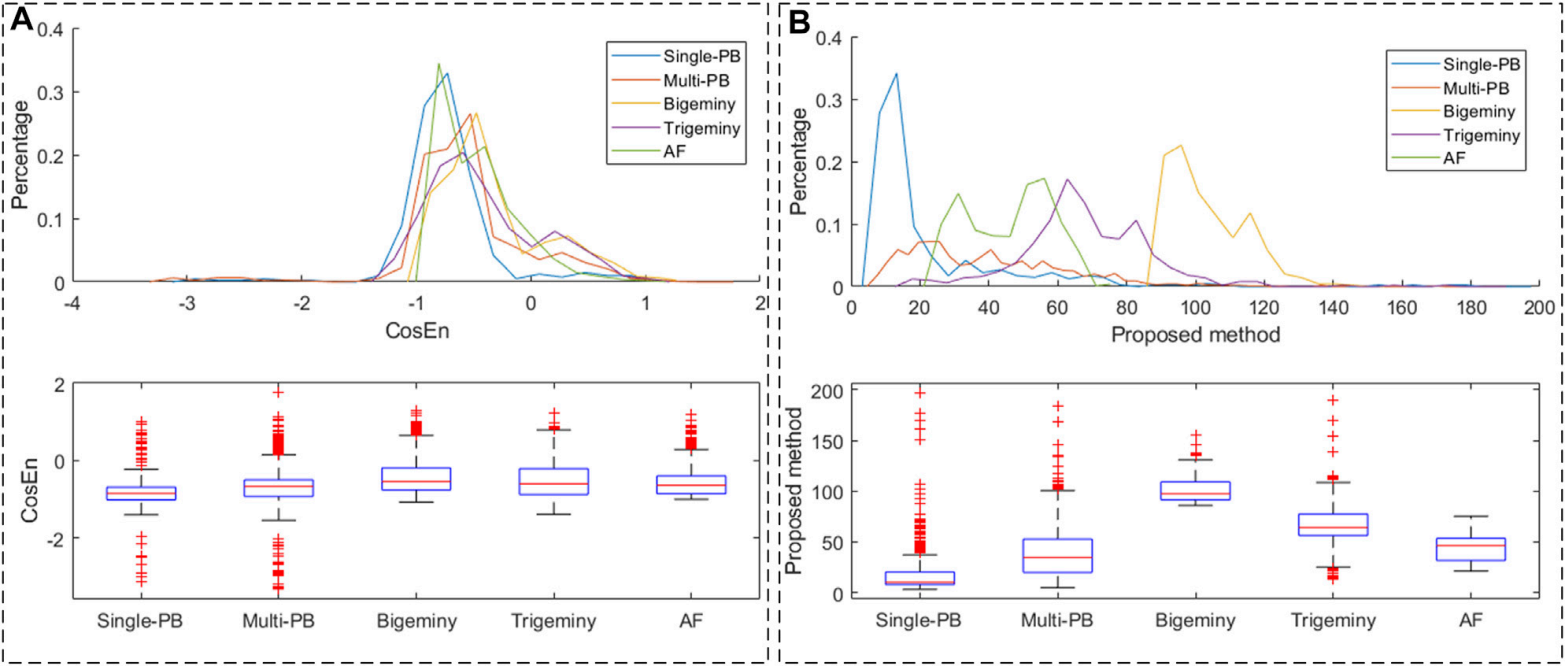
The feature value distribution in ECG segments with PB and AF. **(A)** CosEn; **(B)** *R*_*c*_.

In order to verify the ability of *R*_*c*_ on rejecting premature beats in atrial fibrillation detection, we selected CosEn (15), a common atrial fibrillation monitoring function, for comparative analysis. We tested the *R*_*c*_ and CosEn on the selected 3,000 ECG segments with PB and 3,000 AF segments from MIT-BIH Arrhythmia database. Since the *R*_*c*_ was calculated by 6 RR and there were 30 RR in the test segments, we used the median *R*_*c*_ value of each ECG segments. As shown in Figure 4, the CosEn value distributions of the four types of premature beats and AF segments are approximately the same. While the distribution of *R*_*c*_ values of the four types of premature beats and AF segments was different. Therefore, compared with CosEN, the proposed *R*_*c*_ value is more conducive to eliminating false detections caused by premature beats.

### 5.2 RR-Interval Based Cluster Analysis

**FIGURE 5:**
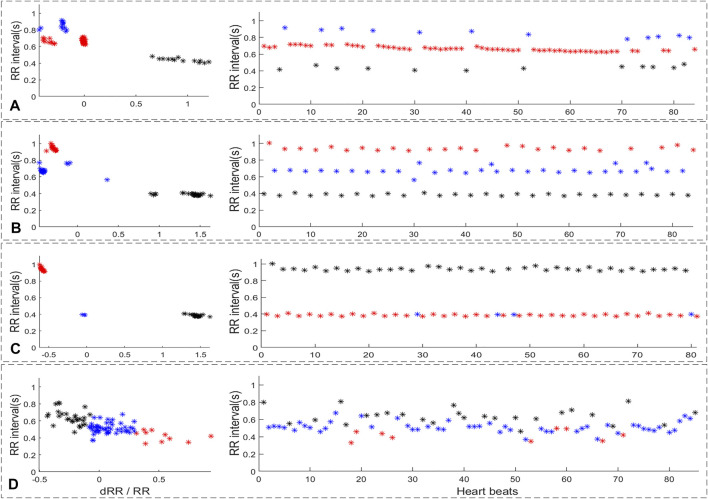
The RR-interval based cluster analysis result of four different rhythm. **(A)**: multi-PB, **(B)**: trigeminy, **(C)**: bigeminy, **(D)**: AF.

Figure 5 shows the RR-interval-based cluster analysis results for four different rhythms. After cluster analysis, the RR interval sequences of ECG segments with multi-PB or trigeminy rhythm were divided mainly according to the numerical value of the RR interval. The mean values of the three types of RR after clustering are quite different. Although the smaller RR interval in the RR sequences of ECG segments with bigeminy were divided into to two classes, the difference between the mean value of the larger RR intervals and the other two categories is sufficiently significant. However, the AF RR intervals of 3 cluster analysis categories did not have clear classification boundaries, and the mean RR of the three categories were nearly equal. Therefore, the possibility of ECG with AF rhythms entering subsequent premature beat reject analysis steps through cluster analysis is negligible.

### 5.3 Premature Beat Reject Method

#### 5.3.1 Single Premature Beat Recheck

**FIGURE 6:**
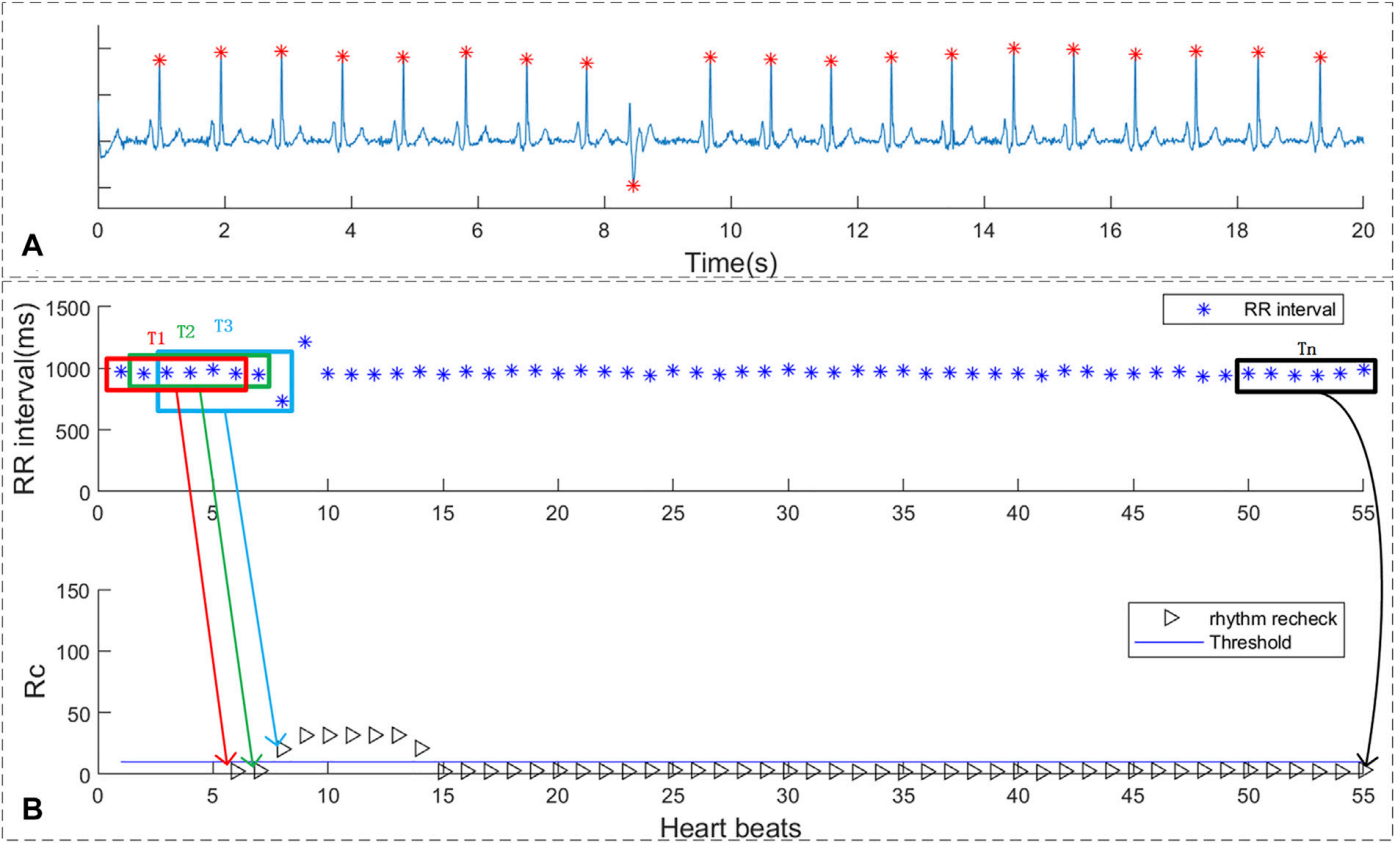
The rhythm recheck result of ECG with single PVC. **(A)** ECG segment with single PVC beats; **(B)** RR intervals and suspicious segment screening result.

As shown in Figure 6, the RR intervals and suspicious segment screening result were shown in sub-figure 6B. The blue straight line represents the threshold, and the black triangle corresponds to the result of the rhythm screen. If the rhythm screen result exceeded threshold, it is considered to be a suspected atrial fibrillation rhythm. It can be concluded that suspicious AF segments screen method indeed consider the rhythm change caused by a single-PB as suspicious AF. However, the duration of the short-term rhythm changes caused by single premature no longer than 10 beats. Thus, the proposed single-PB recheck method can accurate remove the single premature from suspect AF segments.

#### 5.3.2 Multi Premature Beats Recheck

**FIGURE 7:**
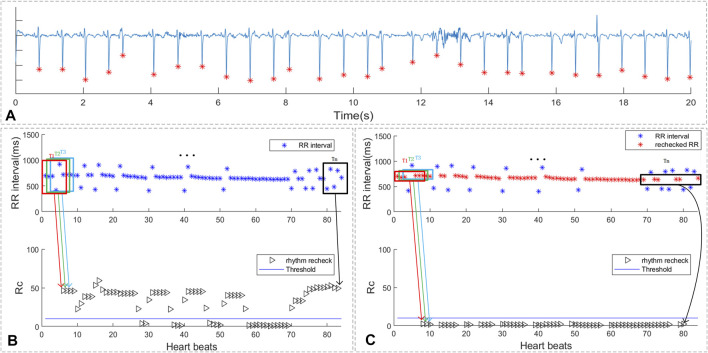
The rhythm recheck result of ECG with multi premature beats. **(A)** ECG segment with multi PAC beats; **(B)** RR intervals and suspicious segment screening result. **(C)** the rechecked RR intervals and suspicious segment screening result

As shown in sub-figure 7A of Figure 7 , there are 4 PAC beats in the 29 beats. In sub-figure 7B, the ECG segment with multi-PB was classified as suspicious AF by the proposed screen method and the duration of rhythm change exceeds 10 beats. As shown in sub-figure 7C the RR intervals around median value of the RR interval sequence were reselected for rhythm screen and were marked as “red *”. The rhythm screen result of the re-selected RR intervals were all below the threshold. Thus, the ECG segments with multi-PB can also be removed by the multi-PB recheck method.

#### 5.3.3 Premature Beat Recheck of Bigeminy and Trigeminy

**FIGURE 8:**
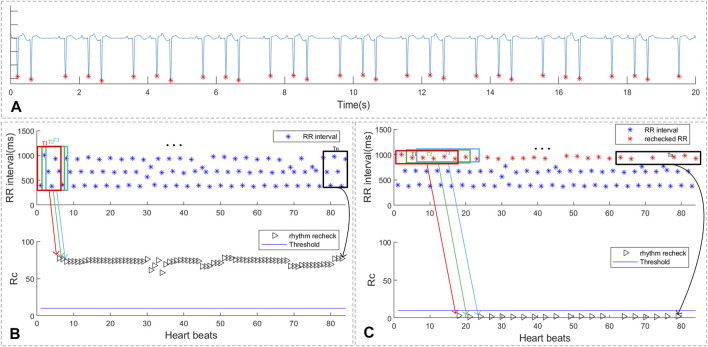
The rhythm recheck result of ECG with trigeminy PVC. **(A)** ECG segment with trigeminy PVC; **(B)** RR intervals and suspicious segment screening result. **(C)** the rechecked RR intervals and suspicious segment screening result

As shown in Figure 8, the ECG segments in sub-figure 8A was ECG with trigeminy premature rhythm. The RR intervals of the ECG segments, which was shown in sub-figure 8B, marked as red and black triangles represent the rhythm scan results of each QRS. The blue line in the sub-figure 8B was the threshold of the rhythm screen method. As shown in sub-figure 8C, the red points refer to the selected larger RR intervals for further recheck while the blue points are the RR intervals with small value and were filtered. The black triangles represent the recheck rhythm screen results of the selected larger RR intervals. It can be concluded that after selecting larger RR intervals, the rechecking rhythm screen result of the trigeminy ECG has been less than the threshold value. Thus, the proposed method can reduce the influence of bigeminy and trigeminy on PAF detection.

### 5.4 PAF Rhythms Detection

The ECG segments with premature beats, which is common in patients with PAF, which is easy to be misjudged as AF. The proposed method is dedicated to eliminating the false alarms caused by ECG with premature beats being misjudged as AF rhythms. Compared with traditional machine learning algorithms, the proposed method does not divide the ECG signal into segments, but evaluates each heartbeat. And finally, we determine the continuous heartbeat segment for PAF. Therefore, even if the proposed method has false alarms, it will behave as a continuous ECG segment, which is convenient for doctors to recheck. The PAF-score was designed to test the overlap ratio of the detection result and the labeled PAF time. Compared with sensitivity and specificity, PAF-score scores each PAF segment, and short paf segment have the same effect on the final score with the long paf segment. As shown in Table 2, the rule-based detection method was utilized to locate the paroxysmal AF of 23 recordings from MIT-AFDB and the average PAF-score was 0.912. The PAF-score of the four recording with most PAF segments were lower than the average score. This is mainly because some of the PAF segments in these recordings only have a short duration, but the duration of detected results are longer, which is resulting in the PAF-scores of these segments lower than 0.5.

In addition, we also count the missed segments and misjudged segments to comprehensively evaluate the performance of PAF detection method. The total number of PAF segments detected was 356, of which 90.73% had PAF ECG. This means that the detected AF segment will increase the workload of the re-examiner by about 10%. Nevertheless, the proposed method achieved 96.43% cove rate (sensitivity) and 97.24% specificity on the 23 records of MIT-AFDB. Thus, although there were some misjudged segments, they only account for 2.76% of non-AF heartbeats. Compared with misjudged segments, the missed segments are relatively fewer, and they are all short duration PAF segments. Therefore, the detection of short duration PAF segments poses a greater challenge to the PAF detection algorithm.

**FIGURE 9:**
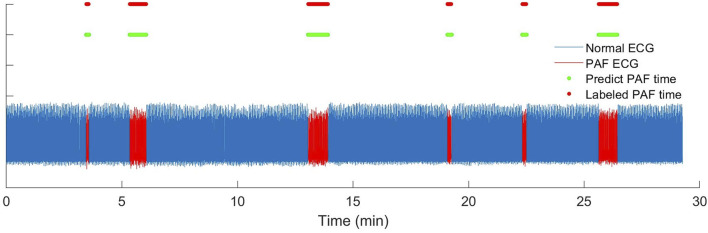
The rhythm recheck result of wearable ECG segments in CPSC 2021

We also test on wearable ECG recordings, and five PAF patients from CPSC 2021 were selected as test ECG recordings. However, the complex noise in the wearable ECG signal which can easily lead to QRS location errors. Therefore, the result of wearable ECG has more misjudgement, and the proposed method obtained an accuracy of 95.74% on the wearable ECG. As shown in Figure 9, the ECG waveform with blue color was normal ECGs and the red ECG waveform were the labeled PAF ECGs. The short line with green color were detected PAF result of the proposed method while the red line was the annotated PAF time. The ECG recording, shown in Figure 8, is one 30-min ECG recording with six PAF segments. There was only very few beats difference between detected PAF results and the labeled result, which indicated that the proposed detection method can effectively locate PAF segment. However, the decrease in accuracy also shows that the proposed method has relatively higher requirements for signal quality.

### 5.5 Limitation

Although this method shows a good performance in detecting PAF, it has certain shortcomings and needs subsequent improvement. The main defects include: 1) This method relies on the accuracy of the QRS detection algorithm. 2) This method may not be suitable for analyzing wearable ECGs with poor signal quality.

## 6 Conclusion

The present study shows that although the proposed PAF detection method is simple, it has good performance in the PAF detection of long-term ECGs. The proposed detection method can effectively eliminate arrhythmias that are easily confused with atrial fibrillation, such as single-PB, multi-PB, premature beat recheck of bigeminy and trigeminy. The proposed model with low computational complexity, and has great potential in the low-complexity analysis of wearable ECG devices.

## Data Availability

The original contributions presented in the study are included in the article/Supplementary Material, further inquiries can be directed to the corresponding author.
